# TCF12 Deficiency Impairs the Proliferation of Glioblastoma Tumor Cells and Improves Survival

**DOI:** 10.3390/cancers15072033

**Published:** 2023-03-29

**Authors:** Yunong Pang, Sichang Zhou, Paul Zumbo, Doron Betel, Babacar Cisse

**Affiliations:** 1 Department of Neurological Surgery, Weill Cornell Medicine, New York, NY 10065, USA; 2 Institute of Computational Biomedicine, Weill Cornell Medicine, New York, NY 10021, USA

**Keywords:** glioblastomas (GBMs), TCF12, cell cycle and proliferation, xenograft, oligodendrocytic lineage

## Abstract

**Simple Summary:**

Human glioblastoma is the most common and malignant primary brain tumor and is universally fatal. Currently, there is no cure for the disease. One major hurdle to developing effective therapies against GBM has been our lack of understanding of regulators of crucial processes and features that define and sustain tumor cells. This study reveals a novel role of transcription factor TCF12 in the regulation of proliferation in GBM tumors using human patient-derived cell lines and an in vivo mouse GBM model. We find that TCF12 deficiency impairs the proliferation of tumor cells in vitro and slows tumor growth in vivo resulting in improved overall survival. We also find that TCF12 regulates the expression of some key regulators of the cell cycle in tumor cells.

**Abstract:**

Isocitrate dehydrogenase (IDH)-wild-type glioblastoma (GBM) is the most common and aggressive primary brain tumor which carries a very poor overall prognosis and is universally fatal. Understanding the transcriptional regulation of the proliferation of GBM tumor cells is critical for developing novel and effective treatments. In this study, we investigate the role of the transcription factor TCF12 in the regulation of GBM proliferation using human and murine GBM cell lines and an in vivo GBM xenograft model. Our study shows that TCF12 deficiency severely impairs proliferation of tumor cells in vitro by disrupting/blocking the G1 to S phase transition. We also discover that TCF12 loss significantly improves animal survival and that TCF12-deficient tumors grow much slower in vivo. Overexpression of TCF12, on the other hand, leads to an increase in the proliferation of tumor cells in vitro and more aggressive tumor progression in vivo. Interestingly, loss of TCF12 leads to upregulation of signature genes of the oligodendrocytic lineage in GBM stem cells, suggesting a role for TCF12 in inhibiting differentiation along the oligodendrocytic lineage. Transcriptomic data also reveals that loss of TCF12 leads to dysregulation of the expression of key genes in the cell cycle. Our work demonstrates critical roles of TCF12 in GBM tumor progression.

## 1. Introduction

Isocitrate dehydrogenase (IDH)-wild-type glioblastoma (GBM) carries a poor overall prognosis and is a universally fatal disease [1]. Current standard of care for newly diagnosed GBM includes surgical resection followed by radiation and chemotherapy; however, the median survival is still only 15 months [2] and recurrence is inevitable. There is significant intratumoral cellular heterogeneity and plasticity in IDH-wild-type GBMs, and this cellular diversity renders them resistant to currently available therapeutic approaches [3,4,5]. Recent work has revealed that IDH-WT GBM cells exist in four main cellular states: astrocyte-like (AC-like), mesenchymal-like (MES-like), oligodendrocyte progenitor cell-like (OPC-like), and neural progenitor cell-like (NPC-like) [6]. Copy number amplifications of the CDK4, EGFR, and PDGFRA loci and mutations in the NF1 locus influence the frequency of cells in NPC-like, AC-like, OPC-like, and MES-like states, respectively [6]. These states can recapitulate distinct cell types, are influenced by the microenvironment, and more importantly, show plasticity [6]. Tumor cells can adapt different states at different times [7]. A critical subset of IDH-WT GBM cells, called glioblastoma stem cells (GSCs), are thought to play critical roles in the initiation of the tumor, generate cellular heterogeneity, confer resistance to therapy, and cause recurrence [8,9]. GSCs can survive and proliferate in hostile microenvironments by overriding normal cell cycle checkpoints that restrict normal stem cells [10,11]. GSCs also demonstrate key stem cell features such as self-renewal, proliferation, and multipotency. Despite our recognition of their critical roles in IDH-WT GBM, not a single GSC-targeted therapy has been developed to date. Efforts to develop such therapies have been hampered by a lack of a thorough understanding of the biology of GSCs [9]. Nevertheless, there have major breakthroughs and progress in our understanding of GSCs since they were discovered a decade ago. Currently, there is not a single aspect of the biology of GSCs that is not being actively and extensively investigated [12].

One particular and crucial area of investigation is the transcriptional regulation by master transcription factors of the different states, processes, pathways, and genetic and epigenetic programs of tumor cells, especially GSCs [13]. Like other cancers where transcriptional signatures of cancer stem cells predict overall patient outcome, it is widely accepted that the same holds true for GSCs [13]. Therefore, a number of studies have characterized the roles of various transcription factors in human GSCs [14,15,16,17]. It has been shown that at least some GSCs are derived from transformed adult neural stem cells (NSCs) in the subventricular zone of the lateral ventricle [14,18]. One of the implications of this fact is the possibility that NSCs and GSCs use similar master transcription factors to maintain key stem cell features and define genetic program. GSCs live in the most hypoxic regions of the tumor and yet they can proliferate independently of exogenous mitogens, and this is critical for tumor progression, resistance to therapy, and recurrence [19,20]. This suggests that cell intrinsic programs and factors regulate the proliferation of GSCs. One key level of regulation is that of transcription factors that regulate various aspects of the cell cycle. Transcriptional regulation of the cell cycle for IDH-WT GSCs is important to tumor progression, resistance to therapy, and recurrence [19]. A thorough understanding of transcriptional regulation of proliferation of IDH-WT GSCs will undoubtedly pave the way for therapeutic inhibition or disruption to help cure, slow down, or/and prevent recurrence of IDH-WT GBMs.

Here, we investigate the role of the transcription factor TCF12 in the regulation of proliferation of IDH-WT GSCs. TCF12 is a member of E proteins or class I basic Helix–Loop–Helix (bHLH) proteins which are known master regulators of various cell types [21,22]. TCF12 has been shown to be involved in the control of proliferating adult NSCs and to play a role in sustaining their undifferentiated state [23,24,25,26]. However, its function in IDH-WT GSCs in unknown. Godoy et al. have shown that silencing of TCF12 in a human GBM cell line, U87, has anti-proliferative effects when the cells are grown as neurospheres or monolayers [27]. However, the in vivo role of TCF12 in IDH-WT GSCs, tumor formation, and progression has yet to be investigated in any human patient-derived lines or in vivo murine GBM models. Using mouse models of human IDH-GBM, we describe a role for TCF12 in the regulation of GSCs, tumor progression, and overall survival.

## 2. Materials and Methods

### 2.1. Mice

Wild type mice were on a C57BL/6 background and maintained in the animal facility of Weill Cornell medical school under specific pathogen-free conditions. Both sexes were used for the analysis. Animals were housed on a 12 h light/dark cycle and were given access to food and water ad libitum.

### 2.2. Cell Culture

GL261 and U251 cell lines were a kind gift of Dr. Jeffery Greenfield. The cells were maintained in DMEM with 10% FBS medium in a 37 °C, 5% CO_2_ incubator. Human-derived GSCs, named the CBTP-14 cell line, were cultured in human neural stem cell culture medium (DMEM/F-12 (Gibco#11320033, Waltham, MA, USA), 1xN-2 (Gibco#17502-048), 1xB-27 (Gibco#17504-044), 20 ng/mL human EGF (Biolegend#585508, San Diego, CA, USA), and 20 ng/mL human FGFb (Biolegend#792508)) in a 37 °C incubator at 5% CO_2_, 2% O_2_.

GL261 stem cells were cultured in neural stem cell culture medium (DMEM/F-12 (Gibco#11320033), 1xN-2 (Gibco#17502-048), 1xB-27 (Gibco#17504-044), 20 ng/mL mouse EGF (Biolegend#585608), and 20 ng/mL mouse FGFb (Biolegend#579606)) in a 37 °C incubator at 5% CO_2_, 2% O_2_.

### 2.3. Western Blot

Cells were lysed in RIPA lysis buffer on ice for 20 min, run through a 10-G needle (BD, San Jose, CA, USA), and spun at 12,000 rpm for 10 min at 4 °C. Protein lysates were quantified via BCA Assay (BioRad, Hercules, CA, USA) and separated on an SDS–PAGE gel. Proteins were transferred onto PVDF membranes (BioRad, Hercules, CA, USA), blocked with 5% milk/TBST, and blotted with primary and secondary antibodies diluted in 5% milk/TBST. Goat anti-rabbit and anti-mouse HRP-conjugated secondary antibodies (Jackson Immuno Research, West Grove, PA, USA) were used. The signal was detected via chemiluminescence using the ECL Prime detection kit (Thermo Fisher Scientific, Waltham, MA, USA). Primary antibodies used were rabbit anti-Tcf12 (D3, SC-28364); mouse anti-CyclinD1 (SC-8396), CyclinD2 (SC-376676), b-Actin (SC-47778) from Santa Cruz Biotechnology (Santa Cruz, CA, USA); rabbit anti-Tcf12(P-21039), -pHH3 (PA5-17869), mouse anti-Ki67 (MA5-14520) from Thermo Fisher Scientific; and mouse anti-Galc (MAB342), rabbit-anti-Oligo2 (AB9610) from Millipore (Burlington, MA, USA).

### 2.4. Histology and Immunofluorescence and Immunohistochemistry

To analyze the histopathology of brain tumors, sections were stained with Hematoxylin and Eosin (Thermo Fisher Scientific) (H&E) according to the protocol from the manufacturer. Images were collected on a Nanozoomer. For immunohistochemical (IHC) analysis, sections were blocked for at least 1 h in 5% BSA (Sigma-Aldrich, Burlington, MA, USA) and 0.3% Triton X-100 (Thermo Fisher Scientific) and incubated overnight at 4 °C in antibody diluted in a blocking solution. Sections were then incubated with a species-specific secondary antibody for 2 h at room temperature (RT). Primary antibodies used were rabbit anti-Tcf12 (D3, SC-28364); Sox2, Nestin, Pax6 are from Millipore.

### 2.5. Quantitative RT-PCR

Total RNA was isolated using Trizol reagent. First, 500 uL Trizol (Invitrogen, Carlsbad, CA, USA) was added to each sample and incubated at room temperature for 5 min; 100 uL chloroform was then added to each sample and shaken for 15 s. Samples were centrifuged at 4 °C, 12,000× *g* for 15 min. After centrifugation, supernatants were transferred to a fresh tube, and 250 uL isopropanol and 1 uL Glycoblue (Invitrogen) were added. Samples were incubated at −20 °C for 3 hours then centrifuged at 4 °C 12,000× *g* for 15 min. Samples were washed twice with 70% EtOH, air dried, and resuspended with TE buffer.

For RT-PCR, 1–2 µg RNA was used with oligo(dT) primers to synthesize cDNA using the SuperScript III First-Strand Synthesis System (Invitrogen). cDNA was diluted five-fold for quantitative PCR. Quantitative real time PCR was performed with the SYBR Green Mastermix (Applied Biosystems, Waltham, MA, USA) on a QuantStudio™ 6 Flex Real-Time PCR System (Applied Biosystems). Relative gene expression was normalized to the GADPH gene. All reactions were done at 60 °C annealing temperature, with an extension time of 15 s for all the primers. Primers used for each gene are listed bellow (sequences are 5′ to 3′):

CCND1_F: GCGTACCCTGACACCAATCTC

CCND1_R: CTCCTCTTCGCACTTCTGCTC

CCND2_F: GAGTGGGAACTGGTAGTGTTG

CCND2_R: CGCACAGAGCGATGAAGGT

CCND3_F: CGAGCCTCCTACTTCCAGTG

CCND3_R: GGACAGGTAGCGATCCAGGT

Ano4_F: AGTTTTCCACGCAGAAGGTG

Ano4_R: CAGCCTCAAGTTCGTCAAAA

Cryab_F: ATGCGTTTGGAGAAGGACAG

Cryab_R: TCCGGTACTTCCTGTGGAAC

Cp_F: GGGACTATGCTTCTGGCACT

Cp_R: TCTTACTAAAGGTGCCATCTGTG

Mbp_F: TGGCCACAGCAAGTACCAT

Mbp_R: CAGGGAGCCATAATGGGTAG

Enpp2_F: GGCGTCAATCTCTGCTTAGG

Enpp2_R: ACACCGACAGTCAGGAGGTC

Aldoc_F: CCCGCTATGCCAGCATCT

Aldoc_R: TCGAGGTATACATGATGGTCACTC

Id3_F: CTCGACCTTCAGGTGGTCCT

Id3_R: TCAGTGGCAAAAGCTCCTCT

### 2.6. EdU Proliferation Assay

Cells were plated at the desired density, grown overnight, and incubated with 10 uM Edu for 2 h before collection. Treated cells were fixed and parabolized before staining as instructed in the Click-iT Edu Alexa Flour 647^TM^ flow cytometry assay kit (Thermo Fisher).

Stained cells were then analyzed by flow cytometry analysis on DAPI (Ex = 450 nm; Em = 355 nm) and APC (Ex = 670 nm; Em = 650 nm) channels. EDU flow cytometric assay and visualization were performed on Symphony A5 (BD, San Jose, CA, USA), and analyzed by Flow software FlowJo (v10.8.1).

### 2.7. Generation of CRISPR-Mediated Knockout GL261, U251, and Human CBTP-14 Cell Lines

sgRNAs targeting the Tcf12 gene or scramble (described below) were designed, amplified, and cloned into the LentiCRISPRv2GFP vector (Addgene #82416, Watertown, MA, USA) that also encodes Cas9-GFP and confers puromycin resistance. VSV-G-pseudotyped lentiviral particles were produced from the vectors using transient transfection in 293T cells using Lipofectamine 3000 (Invitrogen) and Opti-MEM according to the manufacturer’s instructions. GL261, U251, and human CBTP-14 cells were treated with the virus and changed to growing medium at 24 h post-transfection and selected by sorting of GFP or puromycin selection. The resulting population was either analyzed in bulk or cloned by sorting in 96 well plates, and the resulting clones were screened by WB for protein level and by PCR for the targeting region and sequencing.

For GL261 cells:

sgRNAs targeting scramble (sequences are 5′ to 3′)

mScramble_F: CACCGGCGAGGTATTCGGCTCCGCG

mScramble_R: AAACCGCGGAGCCGAATACCTCGCCGGTGC

sgRNAs targeting mouse Tcf12 (sequences are 5′ to 3′)

mTcf12 sgRNA_F: CACCGGAAAACGAGACCAACAACAC

mTcf12 sgRNA_R: AAACGTGTTGTTGGTCTCGTTTTCC

For CBTP-14# and U251 MG cells:

sgRNAs targeting scramble (sequences are 5′ to 3′)

hScramble-sgRNA_F: CACCGGCGAGGTATTCGGCTCCGCG

hScramble-sgRNA_R: AAACCGCGGAGCCGAATACCTCGCCGGTGC

sgRNAs targeting human Tcf12 (sequences are 5′ to 3′).

hTcf12 sgRNA-F: CACCGCCAATGTCCAGCTTTCATCG

hTcf12 sgRNA-R: AAACCGATGAAAGCTGGACATTGGCGGTGC

### 2.8. MRI of Xenografts

Control and Tcf12 KO GL261 cells prepared as described above were injected individually into adult C57BL/6 mice (5 × 10^4^ per mouse) and imaged by the MRI (Magnetic resonance imaging) method described below at 3 and 5 weeks for tumor detection.

MRI imaging: Axial mouse images were acquired using a 2D TurboRARE sequence on a 7T magnet The sequence used a repetition time (TR) of 2570 ms, an effective echo time (TE) of 48 ms (TE of 12 ms with Rare Factor of 10), and 14 averages. Eighteen contiguous slices of 0.5 mm thickness with no gap were acquired for a total scan time of 15 min. The field of view was 20 mm × 20 mm with an acquired 256 × 256 matrix, yielding an in-plane resolution of 0.078 mm × 0.078 mm.

### 2.9. Statistical Analysis

Comparisons between two conditions were performed with the two tailed Student’s *t* test, and comparisons between three or more conditions were performed with one-way ANOVA and Tukey’s multiple comparison test (GraphPad Prism v9.2.0). *p* < 0.05 was considered to be significant. Kaplan–Meier survival curves were generated by Prism GraphPad.

### 2.10. RNA Sequencing and Data Analysis

Four million cells per reaction were used for RNA sequencing. Total RNA extraction was performed as described above by Trizol reagent and eluted with TE buffer. Quality control was performed using an Agilent Bioanalyzer and NanoDrop. RNA samples were reverse transcribed to generate cDNA libraries (done in the Genomics Resources Core Facility, Weill Cornell medical school). RNA sequencing was run on a 10× machine. Raw reads were quality checked with FastQC v0.11.7 (http://www.bioinformatics.babraham.ac.uk/projects/fastqc/ (accessed on 7 January 2018)). Reads were aligned to the mouse (GRCm38.p6) using STAR v2.7.9a1 with default parameters. Gene abundances were calculated with featureCounts v1.6.22 using composite gene models from Gencode release M25 (mouse). Principle component analysis was performed using the plotPCA function from DESeq2 v1.32.04. Differentially expressed genes were determined with DESeq2 using Wald tests (q < 0.01). Gene set enrichment analysis (GSEA) was performed using fgsea v1.18.05 with gene sets from the Broad Institute’s MSigDB collections. For GSEA, genes were ranked by the DESeq2 Wald statistic. Only pathways with an adjusted *p* value < 0.05 were considered enriched. Over-representation testing was performed using clusterProfiler v4.0.58. Variance-stabilized expression heatmaps were generated, with the values centered and scaled by row.

## 3. Results

### 3.1. Tcf12 Is Upregulated in Human GBM Cells and Is Highly Expressed in Enriched GSCs

**Figure 1:**
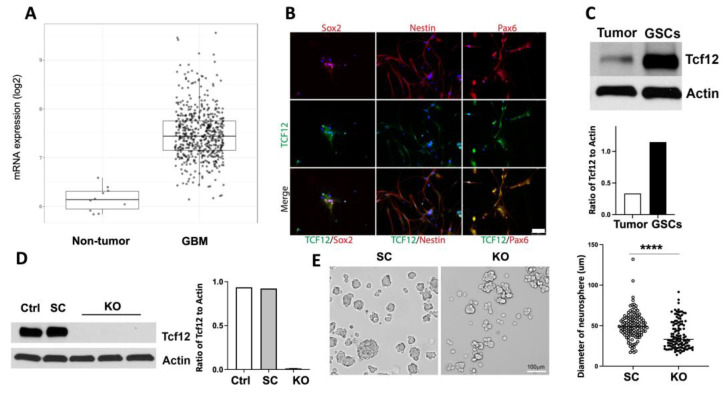
TCF12 is upregulated in human GBM cells and is specifically enriched in GSCs. (**A**) mRNA expression plot shows higher TCF12 expression in human GBM cells compared to normal nontumor cells. Source: http//gliovis.bioinfo.cnio.es. (**B**) Representative immune staining of human glioblastoma stem cells (GSCs) showing co-localization of TCF12 with known stem cell markers Sox2, Nestin, and Pax6. (**C**) Western blot and quantification showing enrichment of TCF12 in human GSCs compared to the original tumor samples from which they were enriched. See also Figure S1A,B. (**D**) Western blot and quantification showing deletion of TCF12 in GL261 using the CRISPR/Cas9 system. These are single knockout clones. Ctrl: control, SC: scramble, KO: knockout. The uncropped blots are shown in Figure S1. (**E**) Bright field pictures showing neurosphere formation by scramble and TCF12 KO GL261 cells. Neurosphere sizes are quantified based on the particle size. ****, *p* < 0.0001. Scale bar: 5 µm in (**B**), 100 µm in (**E**).

To study the role of TCF12 in the proliferation of GSCs, we started by looking at its expression levels in human GBM cells compared to nontumor cells. To this end, we analyzed its expression levels in human GBMs using GlioVis, a web-based tool that helps analyze and explore expression datasets of human brain tumors. We found that TCF12 mRNA was highly upregulated in GBM tumor cells compared to nontumor cells (Figure 1A). We next sought to confirm the expression of TCF12 in human IDH-WT GSCs. To this end, we used tumor samples from human IDH-WT GBM patients within 1–3 h of surgical resection and cultured them in serum-free neural stem cell medium to enrich for GSCs. Two to three weeks after initial culture, most of the live cells expressed known GSC-specific markers such as Sox2 and nestin (Figure 1B). We also quantified TCF12 protein levels by Western blot, and consistently found that they were higher in GSCs compared to the original bulk tumors from which they were enriched (Figure 1C and Figure S1A,B).

### 3.2. TCF12 Regulates the Cell Cycle in Glioblastoma Stem Cells

To gain insight into the functions of TCF12 in GSCs, we attempted to delete it in human IDH-WT GSCs. However, the deletion seemed lethal as no TCF12-deficient human GSCs could survive. Therefore, we turned to the GL261 murine GBM model which is a syngeneic mouse model of GBM in C57BL/6 mice that does not require a deficient immune system [28,29,30]. Injection of the GL261 cells into the brains of mice causes them to develop HGGs spontaneously, and most of the mice die within a few weeks [28,29,30]. Using the CRISPR/Cas9 system, we deleted TCF12 in GL261 tumor cells and selected out single knockout (KO) clones. Deletion of TCF12 was confirmed by Western blot and sequencing (Figure 1D and Figure S1C,D). We then confirmed the presence of GSCs in this model by the neurosphere assay by culturing the scramble and TCF12 KO tumor cells in NSC medium and in a hypoxic environment. Neurospheres formed within 24 h in culture (Figure 1E). Interestingly, TCF12 KO neurospheres were significantly smaller than their SC counterparts.

**Figure 2:**
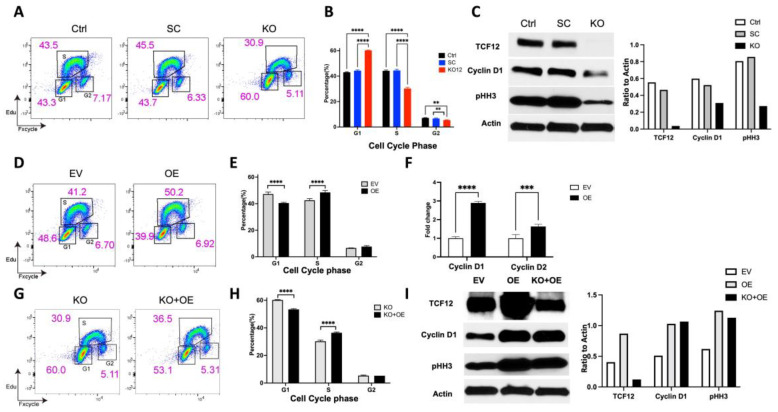
TCF12 regulates the cell cycle in GL261 cells. (**A**) Graphical representation of the flow cytometry data for EdU proliferations assay in Ctrl: control GL261 cells, SC: scramble cells, KO: TCF12 knockout GL261 cells. Cell cycle phases are indicated with the relative cell numbers in each phase. (**B**) Quantification results from A, *n* = 3 per cell type. (**C**) Western blot and quantification showing that complete knockout of TCF12 in GL261 cells causes lower protein levels of a cell cycle gene: cyclin D1; and a mitotic marker: pHH3. Actin is the loading control. (**D**) Graphical representation of the flow cytometry data of EdU proliferation assays for cells that received empty vector (EV), GL261 cells overexpressing TCF12 (OE). (**E**) Quantification results from (**D**), *n* = 3 per cell type. (**F**) qPCR data showing upregulation of key regulators of the cell cycle in TCF12-overexpressing GL261 cells compared to empty vector cells. (**G**) Graphical representation of the flow cytometry data for EdU proliferation assays in TCF12 KO cells (KO) and TCF12 GL261 KO cells that were complemented with TCF12 (KO + OE). (**H**) Quantification results from (**G**), *n* = 3 per cell type. (**I**) Western blot and quantification showing that overexpression of TCF12 restores TCF12 protein and increases the protein levels of cyclin D1 and pHH3. Ctrl: control GL261 cells, SC: scramble cells, KO: TCF12 knockout GL261 cells, EV: GL261 cells that received empty vector, OE: GL261 cells overexpressing TCF12, KO+OE: TCF12 KO GL261 cells in which TCF12 was restored. The uncropped blots (**C**,**I**) are shown in Figure S2. ** *p* < 0.01; *** *p* < 0.001, **** *p* < 0.0001 (**B**,**E**,**F**,**H**).

We next sought to determine the effects of TCF12 loss on the proliferation of GL261 tumor stem cells. Using the EdU proliferation assay, control, scramble, and TCF12 knockout GL261 cells were incubated with EdU for 3 h and then analyzed by flow cytometry. We observed a significant decrease of TCF12 KO tumor cells in the S phase of the cell cycle compared to control or scramble tumor cells (Figure 2A,B). This suggested that TCF12 or/and its target genes may be required for the proper transition from the G1 to the S phases of the cell cycle. To test this hypothesis, we measured the expression levels of cyclin D1 (CCND1), one of the key regulators of the G1 to S phase transition [31].Protein levels of CCND1 were significantly lower in the TCF12 KO tumor cells compared to control and scramble tumor cells (Figure 2C and Figure S2). CCND1 has been shown to be expressed in human GBMs and its expression levels correlate negatively with overall survival of human patients [32]. Phospho-histone 3 (pHH3) is a known marker of mitosis [33], and we observed downregulation of pHH3 in TCF12 KO compared to control and scramble tumor cells (Figure 2C). To further confirm a direct role for TCF12 in the regulation of the cell cycle of GL261 cells, we overexpressed it in GL261 cells and restored it to TCF12 KO GL261 cells (Figure 2I). Using the EdU incorporation assay, we observed that the percentage of TCF12-overexpressing cells (OE) in the S phase was higher than that of control cells with an empty vector (Figure 2D,E). We also observed increased proliferation of TCF12 KO tumor cells to which TCF12 was restored compared to TCF12 KO cells (Figure 2G,H); however, the increase in the proliferation of TCF12 KO cells complemented with TCF12 did not reach baseline control levels (Figure 2H). We next tested if the effects of TCF12 overexpression could rescue the expression levels of cyclin D1 and pHH3. Indeed, TCF12 overexpression led to an increase in the protein levels of cyclin D1 and pHH3 (Figure 2I and Figure S2). Analysis of levels of cyclin D1 and cyclin D2 by qPCR revealed significant upregulation in TCF12-overexpressing cells compared to empty vector control (Figure 2F). These findings suggest that TCF12 regulates the expression of genes that play key roles in the cell cycle and specifically in the G1 to S transition.

**Figure 3:**
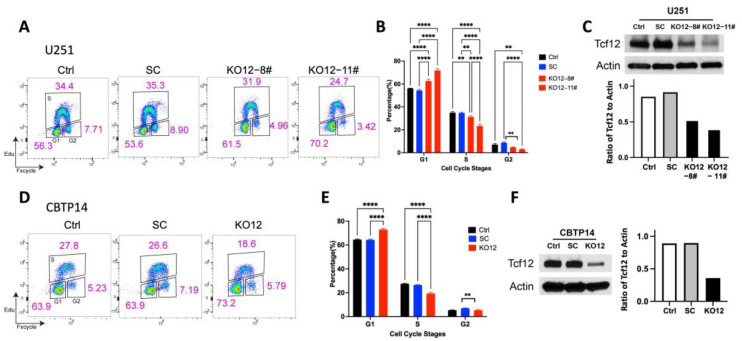
Tcf12 regulates proliferation in the human GBM cell line U251 and patient−derived GSC cells. (**A**) Graphical representation of the flow cytometry for EdU proliferation assays on two TCF12 knockout U251 clones, KO12−8# and KO12−11#, with controls. Cell cycle phases are indicated with the relative cell numbers in each phase. (**B**) Quantitative result from (**A**), *n* = 3 per cell type. (**C**) Western blot showing complete knockout of TCF12 in U251 cells. (**D**) Graphical representation of the flow cytometry of EdU proliferation assays on TCF12 knockout GSC clones of CBTP#14, with controls. (**E**) Quantitative result from (**D**), *n* = 3 per cell type. (**F**) Western blot showing complete knockout of TCF12 in patient-derived GSC CBTP#14 cells. The uncropped blots are shown in Figure S3. ** *p* < 0.01, **** *p* < 0.0001 (**B**,**E**).

We next tested the effects of TCF12 deficiency on the proliferation of human–derived GSCs. To this end, we knocked down TCF12 in the GBM human cell line U251MG and GSCs derived from one of our patients, CBTP#14. As mentioned earlier, our attempts to knock out TCF12 in these GSC cell lines were unsuccessful. However, we were able to perform knockdown. We then performed EdU proliferation assays and found that TCF12 knockdown impaired the proliferation of both U251MG (Figure 3A–C and Figure S3A,B) and CBTP#14 (Figure 3D–F and Figure S3C).

### 3.3. TCF12 Loss Leads to Upregulation of Signature Genes of the Oligodendrocytic Lineage

**Figure 4:**
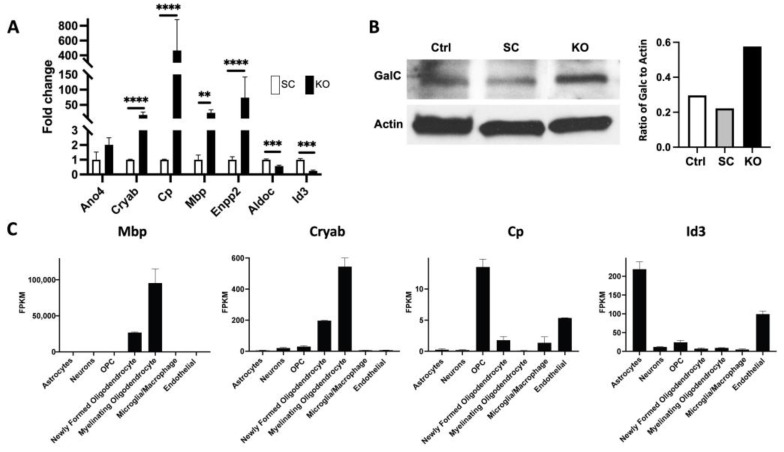
TCF12 loss causes upregulation of oligodendrocyte-specific genes in GL261 cells. (**A**) qPCR validation data showing upregulation of genes enriched in oligodendrocytic lineages: Ano4, Cp, Cryab, Mbp, and Enpp2; and downregulation of genes enriched in astrocytes: Aldoc and Id3. ** *p* < 0.01, *** *p* < 0.001, **** *p* < 0.0001. (**B**) Western blot analysis and quantification showing upregulation of GalC, a marker for mature oligodendrocytes, in TCF12 KO GL261 cells compared to control and scramble cells. The uncropped blots are shown in Figure S4. (**C**) RNA-Seq data from the Brain RNA-seq database (brainrnaseq.org) showing expression profiles in different lineages of the genes Mbp, Cryab, Cp, and Id3.

To determine the effects of TCF12 on differentiation potential, we evaluated, by qPCR, the expression levels of some signature genes of the neuronal, astrocytic, and oligodendrocytic lineages in TCF12 KO and control GL261 cells. While some known neuronal and astrocytic markers showed downregulation or showed no significant differential expression between TCF12 KO and control cells (Figure 4A), we observed significant upregulation of the oligodendrocyte-specific genes Ano4, Cryab, Cp, Mbp, and Enpp2 in TCF12 KO cells compared to control cells (Figure 4A). Analysis of the Brain RNA-Seq database, brainrnaseq.org, confirmed that these genes are largely specific to oligodendrocytes at various stages of their development and maturation (Figure 4C). We also assessed, by Western blot, the expression levels of GalC, another known and specific maker of mature oligodendrocytes. We found that GalC was significantly upregulated in TCF12 KO GL261 cells compared to WT and SC cells (Figure 4B and Figure S4). This interesting finding coupled with the observed small neurospheres in the TCF12 KO cells suggests a role for TCF12 in the maintenance of stemness and multipotency in GSCs. Moreover, our data suggest that downregulation of TCF12 leads to differentiation towards the oligodendrocytic lineage.

### 3.4. TCF12 Loss Slows Tumor Growth In Vivo and Improves Animal Survival

**Figure 5:**
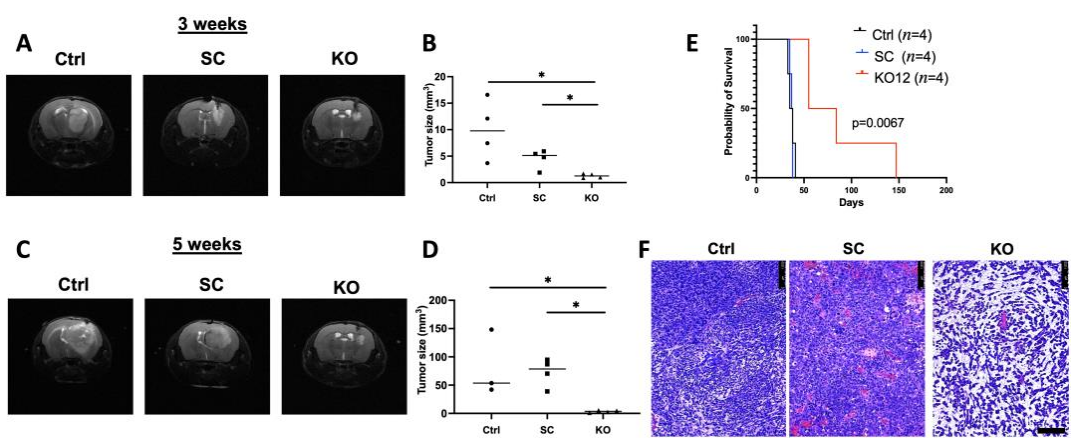
Deletion of TCF12 delays tumor growth in vivo. (**A**) T2 coronal MRI images of whole brain at 3 weeks post-injection of control GL261 tumor cells (Ctrl), scramble GL261 cells (SC), and TCF12 KO cells (KO). (**B**) Tumor sizes measured on MRIs at 3 weeks post-injection of Ctrl, SC and TCF12 KO GL261 tumor-bearing mice. (**C**) T2 coronal MRI images of whole brain at 5 weeks post-injection of Ctrl, SC, and TCF12KO GL261 tumor-bearing mice. (**D**) Tumor sizes were measured on MRIs at 5 weeks post-injection of control, scramble, and TCF12 KO tumor-bearing mice. (**E**) Kaplan–Meier survival curve of mice that were injected with Ctrl, SC, and TCF12KO GL261 cells. (**F**) H&E staining of in vivo tumors after euthanasia, scale bar 1000 μm. * *p* < 0.05 (**B**,**D**). Circles mice recipient of scramble tumor cells; triangles: mice recipient of TCF12 Ko tumor cells.

The results described above demonstrate a clear role of TCF12 in the regulation of the cell cycle and possibly differentiation potential of tumor cells. To gain insight into the effects of TCF12 loss on tumor growth and progression in vivo, and overall survival, we performed intracranial injection of TCF12-competent and TCF12-deficient GL261 cells in mice. To this end, we injected 50,000 cells from each of control, scramble and TCF12 KO GL261 cells into immunocompetent B6 mice (four mice in each group) and monitored tumor initiation and progression by serial MRIs. Three weeks after intracranial injection of tumor cells, mice recipient of control and scramble cells developed significantly larger tumors (Figure 5A,B) than mice that were injected with TCF12 KO cells (Figure 5C,D). We then performed another round of imaging with MRIs five weeks after the initial injections. These MRIs showed significant interval growth of tumors in mice injected with control and scramble tumor cells (Figure 5C,D); however, mice that received TCF12 KO cells showed much less growth during that interval (Figure 5C,D). There was also overall better survival in the TCF12 KO tumor-bearing mice compared to mice injected with control and scramble cells (Figure 5E). Histologic analysis of the tumors showed unusual tumor histology (Figure 5F), noting the prominent mucinous extracellular matrix in TCF12 KO tumors [34].

### 3.5. Overexpression of TCF12 Leads to More Aggressive Tumors In Vivo

**Figure 6:**
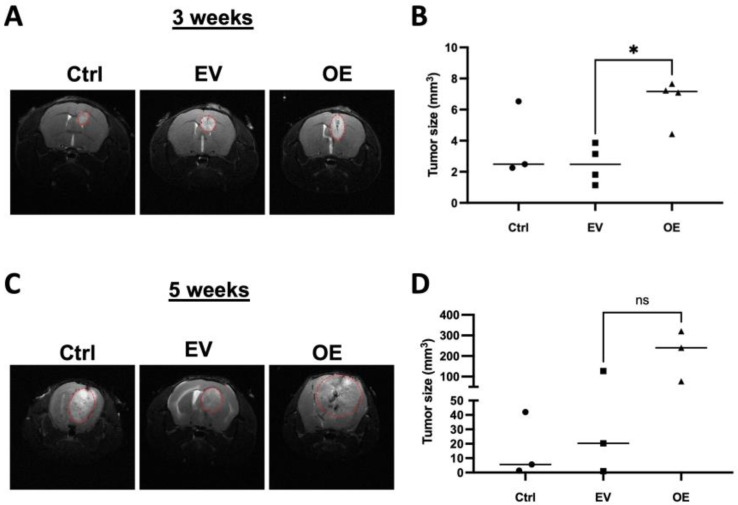
Overexpression of TCF12 in tumor cells leads to more aggressive tumors in vivo. (**A**) T2 coronal MRI images of whole brain at 3 weeks post-injection of mice that were injected with control GL261 cells (Ctrl), GL261 cells that received empty vector (EV), GL261 cells overexpressing TCF12 (OE). (**B**) Tumor sizes measured on MRIs of whole brain at 3 weeks post-injection of mice injected with Ctrl, EV, and OE GL261 cells. (**C**) T2 coronal MRI images of tumor mice at 5 weeks post-injection of control, scramble, and TCF12 KO GL261 tumor bearing mice. (**D**) Tumor sizes measured on MRIs at 5 weeks post-injection of Ctrl, EV, and OE GL261 tumor bearing mice. ns not significant; * *p* < 0.05 (**B**,**D**). Circles: mice recipient of scramble tumor cells; triangles: mice recipient of TCF12 Ko tumor cells.

Based on the in vitro and in vivo results presented, we next sought to determine if overexpression of TCF12 would lead to more aggressive tumors in vivo given the increase in proliferation that we observed in vitro. To test this, we performed intracranial injections of 50,000 cells of either TCF12-overexpressing vector (OE) or empty vector (EV) GL261 cells into immunocompetent mice and monitored tumor appearance by MRI. At three weeks post-injection, TCF12-overexpressing tumors were significantly bigger in size than empty vector control tumors (Figure 6A,B). We repeated the MRIs two weeks after the initial MRIs and we observed larger tumors in the mice that were injected with TCF12-overexpressing tumor cells compared to controls (Figure 6C,D). These results further substantiated the role of TCF12 in the regulation of the proliferation of tumor cells in vitro and in vivo.

### 3.6. Identification of Genes and Pathways Regulated by TCF12 in GBM Cells

**Figure 7:**
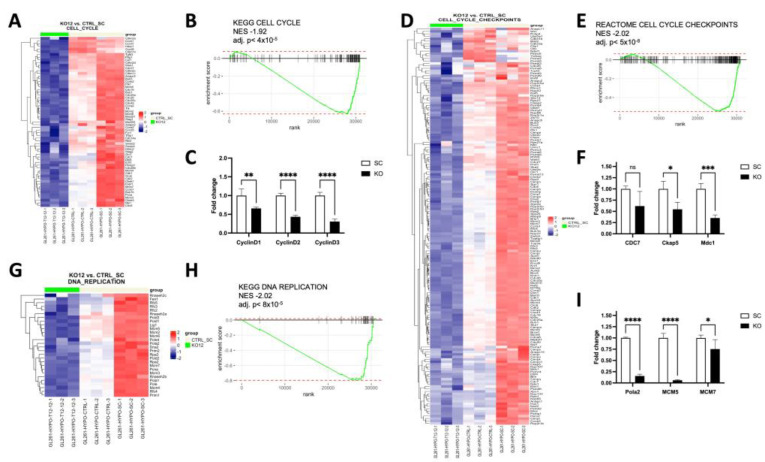
TCF12 loss globally affects critical cellular processes in tumor cells. (**A**) Heatmap showing differential expression of key genes in the cell cycle in TCF12 KO versus SC GL261 cells. (**B**) Representative KEGG enrichment plot for cell cycle genes showing global downregulation of some key regulators and effectors of the cell cycle. (**C**) qPCR data showing validation of the differential expression data for Cyclin D1, Cyclin D2, and Cyclin D3. (**D**) Heatmap showing differential expression of key genes in the cell cycle checkpoint. (**E**) Representative Reactome enrichment plot for cell cycle checkpoints showing global downregulation of key genes involved in cell cycle checkpoints. (**F**) qPCR data showing validation of the differential expression data for Ckap5, Mdc1, and Cdc7. (**G**) Heatmap showing differential expression of regulation of DNA replication. (**H**) Representative Reactome enrichment plot for DNA replication showing global downregulation of key genes involved in DNA replication. (**I**) qPCR data showing validation of the differential expression data for Pola2, Mcm5, and Mcm7. ns not significant; * *p* < 0.05, ** *p* < 0.01, *** *p* < 0.001, **** *p* < 0.0001 (**C**,**F**,**I**).

To gain insight into the genes and pathways that are directly or indirectly regulated by TCF12, we performed transcriptomic profiling of control, scramble, and TCF12 KO GL261 cells by bulk RNA sequencing (RNA-Seq). Differential expression analysis and gene set enrichment analysis (GSEA) showed downregulation of genes involved in the cell cycle (Figure 7A,B), cell cycle checkpoints (Figure 7D,E), and DNA replication (Figure 7G,H). We confirmed the differential expression of a select number of genes by qPCR. These genes included some of the cell cycle genes such as Ccnd1, Ccnd2, and Ccnd3 (Figure 7C); cell cycle checkpoints genes such as Cdc7, Ckap5, and Mdc1 (Figure 7F); and DNA replication genes such as Pola2, Mcm5, and PCNA (Figure 7I). Over-representation analyses (ORA) also demonstrated downregulation of cell cycle and DNA replication genes and enrichment of genes in collagen metabolic processes and extracellular matrix organization.

## 4. Discussion

The work presented here reveals a novel key role for the transcription factor TCF12 in the regulation of proliferation of GBM cells and tumor progression. We found that TCF12 is upregulated in human GBM cells compared to nontumor cells. We also found that TCF12 is highly enriched in human GSCs. Using the murine GBM model, GL261, and human GBM cells, we demonstrate that TCF12 loss severely impairs the proliferation of tumor cells in vitro. Detailed analysis of the cell cycle revealed that TCF12 seems to play a key role in the transition from G1 to S phases of the cell cycle. Deletion of TCF12 resulted in partial block or impairment of the transition from G1 to S phases and decreased proliferation. We found that some key regulators of the cell cycle and markers of mitosis were significantly downregulated in the TCF12 KO tumor cells. To further prove a direct role for TCF12 in the regulation of proliferation of tumor cells, we overexpressed TCF12 in GL261 tumor cells and restored it to TCF12 KO GL261 tumor cells. Overexpression in tumor cells caused increased proliferative activity compared to controls. The restoration of TCF12 to TCF12 KO tumor cells also increased their proliferation although to levels that did not reach baseline control levels. Overexpression or restoration of TCF12 caused upregulation of the same genes involved in the cell cycle and mitosis that were downregulated upon TCF12 loss. This clearly demonstrates a role for TCF12 in the regulation of the cell cycle in GBM tumor cells. The in vivo relevance of these findings was confirmed using a GL261-exograft mouse model using TCF12 KO and control cells. TCF12 KO tumors grew much slower and the mice bearing them lived much longer than control and scramble tumor-bearing mice. We observed the opposite effects when we injected TCF12 OE tumor cells into immunocompetent mice. These tumors grew much faster and the mice died sooner than controls. Histologic analysis of the TCF12 KO tumors revealed an extracellular matrix that is very reminiscent of human oligodendroglioma. Interestingly, it has been shown that TCF12 is mutated in a fraction of human anaplastic oligodendrogliomas [35]. This is rather interesting especially given the fact that we observed upregulation of signature genes of the oligodendrocytic lineage upon TCF12 loss. Moreover, over-representation analysis of RNA-Seq data showed that TCF12 KO cells were enriched for pathways involved in biosynthesis and metabolism of collagen and organization of the extracellular matrix and structure. It will particularly be interesting to look at the role of collagen fibers in the TCF12 KO tumors in delaying tumor growth. Another point is that we cannot denote our TCF12 KO tumors as oligodendrogliomas given the strict requirement for 1p19 co-deletion for oligodendrogliomas by the World Health Organization classification [36]. Nevertheless, our data strongly suggest that TCF12 may play an additional role in maintaining multipotency of tumor stem cells and specifically inhibit differentiation towards oligodendrocytes. It has been shown that TCF12 plays a role in maintaining normal aNSCs in their undifferentiated states [23,24,25,26]. In this study, we showed, by the neurosphere assay, that GL261 does have tumor stem cells. Indeed, TCF12 KO neurospheres were smaller in size than their control counterparts (Figure 1E). Although this is beyond the scope of the work presented here, it remains to be shown whether TCF12 blocks differentiation of normal aNSCs into oligodendrocytes. Our global transcriptomic profiling did reveal relevant genes and pathways that clearly explain the observed phenotypes in vitro and in vivo. Unfortunately, we attempted ChIP-Seq experiments using an anti-TCF12 antibody but could not detect any reliable peaks. Nevertheless, our in vivo and in vitro data coupled with the transcriptomic data clearly demonstrate a role for TCF12 in the regulation of proliferation of tumor cells. This work contributes to our understanding of the transcriptional regulation of the proliferation of GBM cells. While it is difficult to therapeutically target transcription factors, they drive genetic and epigenetic programs in tumor cells; and the genes that they regulate may serve as therapeutic targets. Therefore, elucidation of the transcriptional machineries that regulate key processes and pathways in tumor cells especially tumor stem cells will pave the way for the discovery and development of novel and effective therapies against glioblastoma.

## 5. Conclusions

Glioblastoma is a universally fatal disease and its tumor cells are heterogeneous and plastic. However, abnormal proliferation is their defining feature. Therefore, understanding the regulation of proliferation in GBM tumors is required for the development of effective therapies against GBM. In this study, we reveal a new role for TCF12 in the regulation of the proliferation of GBM tumor cells from in vitro and in vivo data. Although it is limited in scope, our study will hopefully contribute to our understanding of the complex transcriptional machineries that govern and regulate the proliferation of tumor cells.

## Data Availability

The datasets analyzed during the current study are available from the corresponding author upon reasonable request and IRB approval.

## Supplementary Materials

The following supporting information can be downloaded at: https://www.mdpi.com/article/10.3390/cancers15072033/s1, Figure S1. TCF12 is upregulated in human GBM cells and is specifically enriched in GSCs. Figure S2. TCF12 regulates the cell cycle in GL261 cells. Figure S3. TCF12 regulates proliferation in the human GBM cell line U251 and patient-derived GSC cells. Figure S4. TCF12 loss causes upregulation of GalC in GL261 cells.
