# Supplementary material for: TCF12 Deficiency Impairs the Proliferation of Glioblastoma Tumor Cells and Improves Survival
Source: Cancers (Basel). 2023 Mar 29;15(7):2033. doi: 10.3390/cancers15072033 (PMC10093168; doi:10.3390/cancers15072033)
Supplement: Supplementary file 1 [file cancers-15-02033-s001.zip › cancers-2290838-supplementary.pdf]

Supplementary Materials

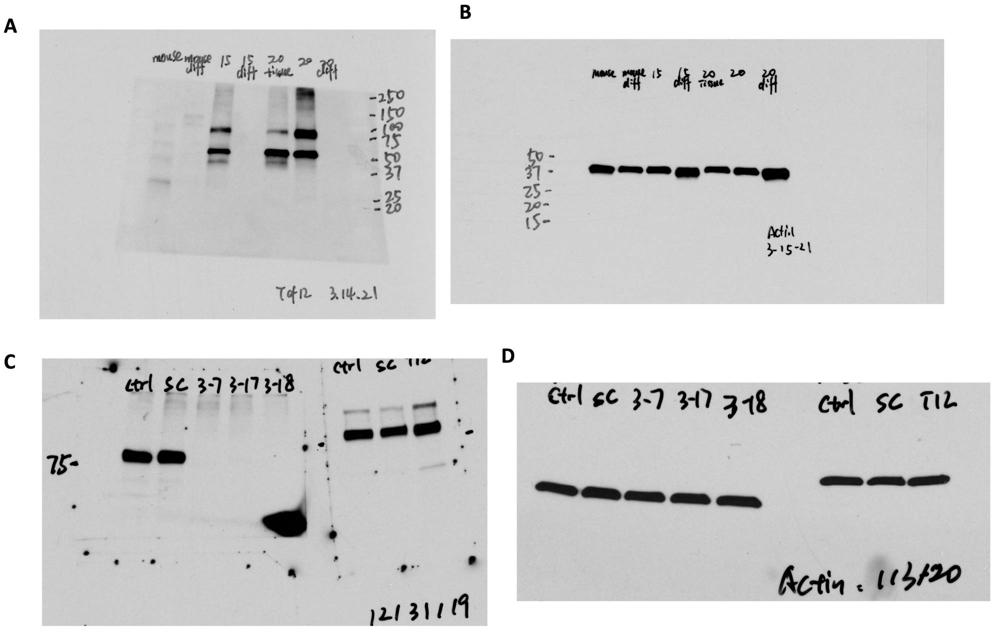

Supplementary Figure S1. TCF12 is upregulated in human GBM cells and is specifically enriched in GSCs. A, B Western blot uncropped images showing enrichment of TCF12 (75KDa) in GSCs. C, D Western blot uncropped images showing TCF12 protein is not detectable in 3 single clones. Actin as the control (B, D).

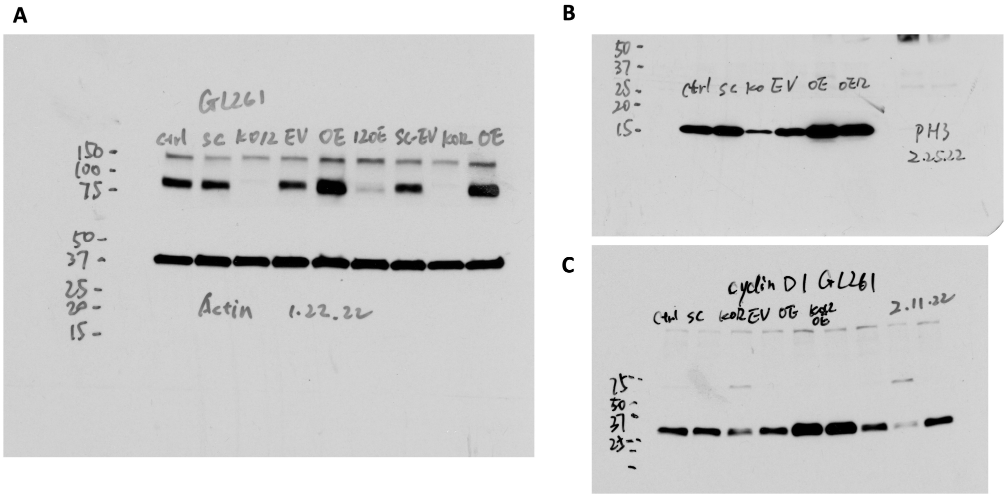

Supplementary Figure S2. TCF12 regulates the cell cycle in GL261 cells. Western blot uncropped images showing the expression level of TCF12 and cell cycle genes Cyclin D1, pHH3 in TCF12KO, and overexpressed cells, with Actin as the control. Ctrl: control GL261 cells, SC: scramble cells, KO: TCF12 knockout GL261 cells. EV: GL261 cells that

received empty vector, OE: GL261 cells overexpressing TCF12, KO+OE: TCF12-GL261 KO cells in which TCF12 was restored.

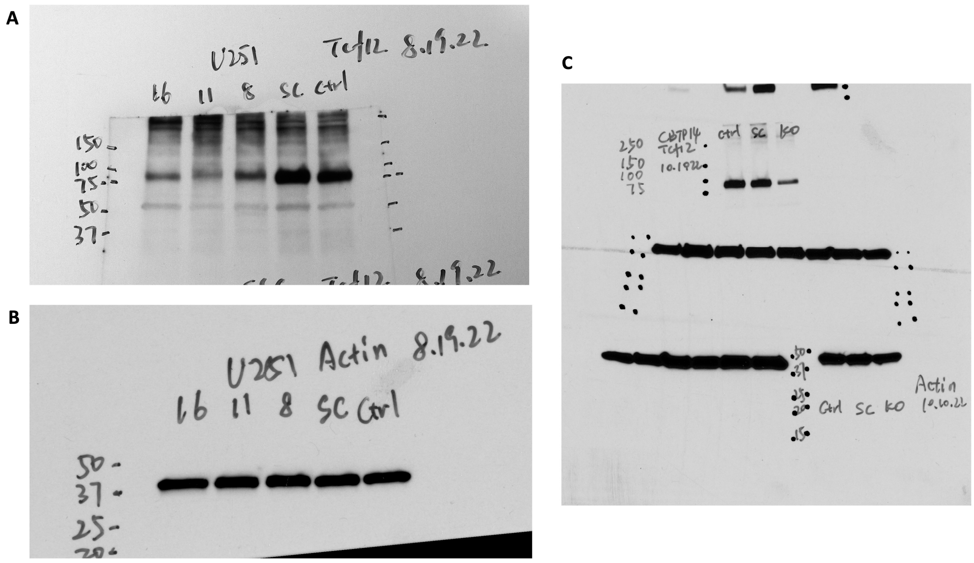

Supplementary Figure S3. Tcf12 regulates proliferation in human GBM cell line U251 and patient-derived GSC cells. Western blot uncropped images showing the knockdown expression of TCF12 in U251 (A,B, 3 clones) and CBTP14 cells, with Actin as the control (B, C).

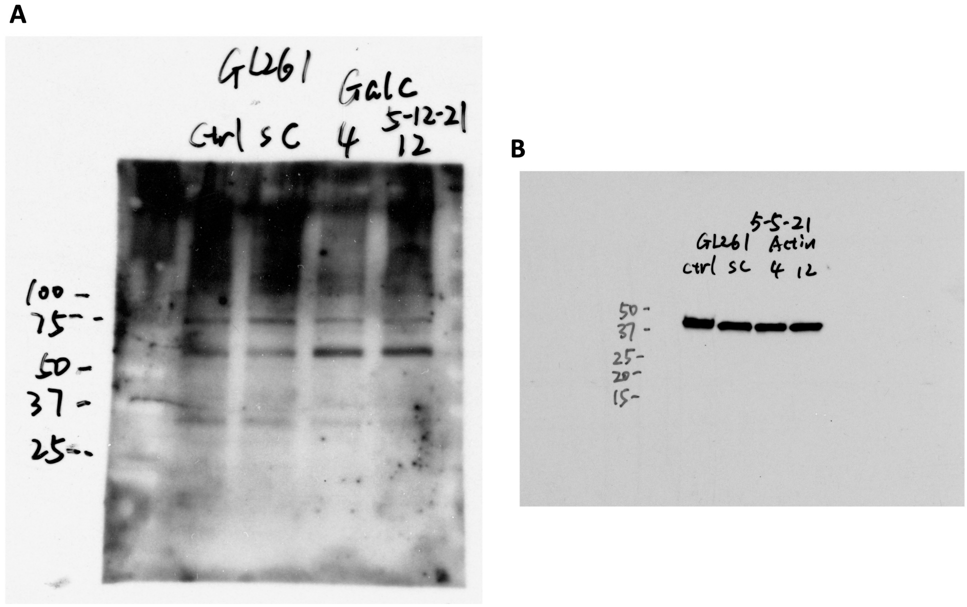

Supplementary Figure S4. TCF12 loss causes upregulation of GalC in GL261 cells. Western blot uncropped images showing expression of GalC (A) in Ctrl, SC and TCF12 knockout clones GL261 cells, with Actin as the control (B).
